# Konjac Glucomannan: An Emerging Specialty Medical Food to Aid in the Treatment of Type 2 Diabetes Mellitus

**DOI:** 10.3390/foods12020363

**Published:** 2023-01-12

**Authors:** Yimeng Fang, Jiahui Ma, Pengyu Lei, Lei Wang, Junying Qu, Jing Zhao, Fan Liu, Xiaoqing Yan, Wei Wu, Libo Jin, Hao Ji, Da Sun

**Affiliations:** 1 Institute of Life Sciences & Biomedical Collaborative Innovation Center of Zhejiang Province, Wenzhou University, Wenzhou 325000, China; 2 Chinese–American Research Institute for Diabetic Complications, School of Pharmaceutical Sciences, Wenzhou Medical University, Wenzhou 325000, China; 3 Key Laboratory for Biorheological Science and Technology of Ministry of Education, State and Local Joint Engineering Laboratory for Vascular Implants, Bioengineering College of Chongqing University, Chongqing 400044, China; 4 Wenzhou City and Wenzhou OuTai Medical Laboratory Co., Ltd. Joint Doctoral Innovation Station, Wenzhou Association for Science and Technology, Wenzhou 325000, China; 5 Wenzhou City and Kunlong Technology Co., Ltd. Joint Doctoral Innovation Station, Wenzhou Association for Science and Technology, Wenzhou 325000, China

**Keywords:** konjac glucomannan, type 2 diabetes mellitus, FSMP, diet therapy, biomedical function

## Abstract

There are many factors causing T2DM; thus, it is difficult to prevent and cure it with conventional treatment. In order to realize the continuous intervention of T2DM, the treatment strategy of combining diet therapy and traditional medication came into being. As a natural product with the concept of being healthy, konjac flour and its derivatives are popular with the public. Its main component, Konjac glucomannan (KGM), can not only be applied as a food additive, which greatly improves the taste and flavor of food and extends the shelf life of food but also occupies an important role in T2DM. KGM can extend gastric emptying time, increase satiety, and promote liver glycogen synthesis, and also has the potential to improve intestinal flora and the metabolic system through a variety of molecular pathways in order to positively regulate oxidative stress and immune inflammation, and protect the liver and kidneys. In order to establish the theoretical justification for the adjunctive treatment of T2DM, we have outlined the physicochemical features of KGM in this article, emphasizing the advantages of KGM as a meal for special medical purposes of T2DM.

## 1. Introduction

Type 2 diabetes mellitus (T2DM) is a metabolic syndrome characterized by decreased insulin sensitivity and insufficient insulin secretion, which is related to the unhealthy metabolism of sugar, fat, amino acids, water, and electrolytes. Genetics [1,2], insulin abnormalities [3], and metabolic disturbance are key factors contributing to the disease. Nowadays, studies have shown that the gut microbiome may also influence the development of T2DM [4,5]. More than 422 million people worldwide have been diagnosed with diabetes mellitus, and the disease claims more than 1.6 million lives annually, according to a recent World Health Organization report [6]. T2DM will affect 439 million people worldwide by the year 2030, making up about 90% to 95% of all cases [7,8]. However, traditional treatment cannot cure it.

T2DM is treated with drugs (such as biguanides, glucosidase inhibitors, and thia-zolidinediones) that manage blood pressure, cholesterol, and blood glucose levels [9]. However, long-term use of pharmaceuticals can lead to adverse effects such as bloating and diarrhea [10], and most individuals with T2DM will eventually require insulin therapy to maintain normal blood sugar levels. In this way, the idea of homology between medicine and food is gradually rising, such as control diets to alleviate the progressive worsening of diabetes [11]. The combination of diet and medicine can control the levels of blood sugar and urine sugar without or with only minimal medicine, especially for patients with mild illness, while those with moderate or severe illnesses even can reduce the amount of medication they require. Among the many therapeutic options studied, there is a growing interest in exploring the therapeutic effects of dietary fiber as a special medical food [12]. Consuming dietary fiber has a multitude of metabolic benefits that are unrelated to changes in weight, including impacts on various metabolic and inflammatory diseases, enhanced insulin sensitivity, and regulation of certain gut hormones. Konjac glucomannan (KGM) is a kind of dietary fiber derived from konjac, that is not easily hydrolyzed by human digestive tract enzymes, and can directly enter the colon and be used by intestinal microorganisms [13]. Long-term consumption can also reduce calories, balance body salt, and improve obesity and diabetes, to improve the sub-health of the human body [14].

**Figure 1:**
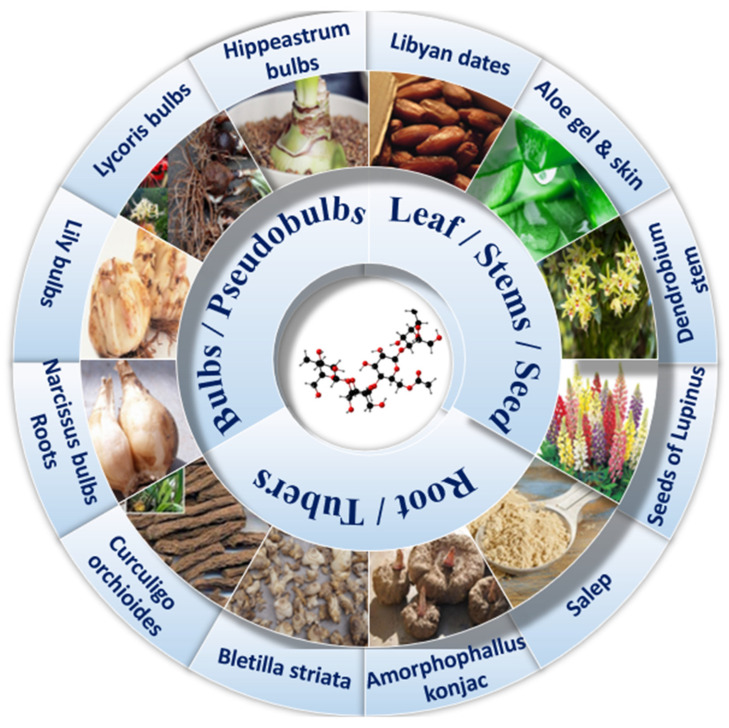
Glucomannan from different sources.

Studies have shown that KGM as a sticky dietary fiber can improve subjective satiety and reduce appetite, which is very beneficial for patients with T2DM [15]. Adding KGM as a dietary additive to noodles can not only slow down the aging rate of noodles, but also improve the storage stability of cooked noodles [16]. This diet may have potential long-term health outcomes, such as lower blood lipids, postprandial blood glucose and insulin levels, and the benefits of this blood sugar response are highly dependent on the type of substrate and the dose of KGM [17,18]. Compared with other dietary fibers (cereal fiber and vegetable fiber), the purified KGM has a single component and is safer for the human body. The amount of dietary fiber per 100 g of other diets (such as soybeans, wheat, and corn) is much lower than that of KGM. In addition, KGM has a wide range of sources (Figure 1), simple extraction process, high efficiency, and considerable price advantages. The most important thing is that in addition to regulating blood sugar, it can also regulate blood lipids, intestinal flora, oxidative stress, immune suppression and so on. However, it should be noted that dietary fiber itself does not have high nutritional value and cannot be used as a long-term staple food. Although KGM cannot be used as a drug in the treatment of disease, it can be used as a special medical food to assist the treatment of type 2 diabetes mellitus. Since increasing evidence linking the consumption of highly processed foods with an increased risk of noncommunicable diseases poses a public health challenge [19,20,21], here we review the structure and physicochemical properties of naturally extracted KGM, discuss its benefits on various physiological and biochemical indexes of type 2 diabetes mellitus, and explain the effects of related mechanisms and pathways, in order to reveal its great potential in food for a special medical purpose (FSMP).

## 2. Extraction and Purification of KGM

Amorphophallus is a perennial herb of Araceae. It has up to 170 varieties, mainly distributed in Southeast Asia and Africa. These varieties are perennial plants with an underground stem in the form of a corm and a highly dissected umbrella-shaped leaf blade [22]. Konjac has a history of more than 2000 years and is a very popular food in China and Japan. Although konjac is poisonous and has a special smell, it is often called “fishy smell”. However, further processing and processing can solve this problem perfectly well. Moreover, the smaller the fishy smell, the higher the purity and the better the quality. The specific processing process is as follows.

KGM is usually obtained from natural high-quality konjac without pesticides and fertilizers (mainly 3 years old, weighing 300–1500 g), extracted by a series of broken walls and refined by multi-stage purification [23,24]. The KGM content in fresh konjac tubers is about 30%, and the highest content of refined konjac flour is 96.9% [25]. From the structure of konjac corm, KGM is mainly distributed in the storage parenchyma below the epidermis of konjac, where a large number of idioblast (also known as cystic cells) and ordinary cells are uniformly dispersed [26]. Ordinary cells contain starch, cellulose, and other components, and the texture is more brittle and very easy to break into dust. However, KGM only exists in idioblast with high hardness, good toughness, and is not easy to break [26]. Although there is a certain amount of crude protein, cellulose, and mineral elements in idioblast, the purity of KGM is enough to meet its medical and food applications.

**Figure 2:**
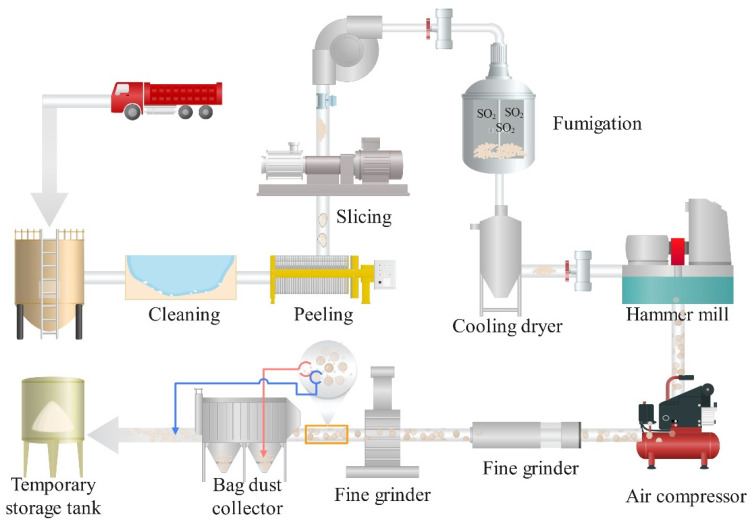
Flow chart of dry preparation of KGM in industry. After washing, peeling, and slicing, the konjac chips will be mixed with SO_2_ and hot air in a special device, and the color will be determined by fumigation, then it will be put into the drying equipment immediately, and the konjac chips will be crushed to the powder by hammer grinder and fine grinder. Finally, under the action of grinding and sorting machine and bag dust collector, the finer flying powder such as starch and cellulose will be removed, and only KGM will be retained.

At present, there are mainly two ways of dry processing and wet processing in industry. The steps of dry processing include washing, peeling, slicing, fixing, drying, grinding and screening of konjac (Figure 2) [27]. In principle, according to the differences in composition, toughness, and hardness between idioblast and ordinary cells, ordinary cells are broken first by mechanical crushing, in which starch, cellulose, and other impurities are gradually crushed into konjac fly powder, while idioblast will not be broken under general crushing conditions, still maintaining the integrity of the particles. Through high-intensity repeated collision and friction, the impurities on the surface of the particles will continue to detach, and then be removed by sieving or wind separation, leaving translucent konjac flour particles. Usually, the KGM treated in this way has a slight smell, but when added to food or water, the smell can also be well masked. Therefore, most people can accept it.

The wet processing principle of KGM is similar to that of the dry process, except that the wet process uses a liquid medium when it is subjected to various mechanical forces such as shear, impact, and extrusion. Its advantage is that in the process of contact between konjac and liquid medium, the soluble impurities in idioblast will gradually dissolve out and then be removed by solid–liquid separation, leaving only glucomannan particles with higher purity. However, KGM is easy to swell and agglomerate in the presence of water, so some blocking solvents (such as ethanol and isopropanol) need to be added. For example, ethanol precipitation is the most commonly used method to obtain refined KGM in the laboratory [28]. Compared with dry processing, wet processing has the advantages of effective impurity removal, higher yield, and better viscosity. It has absolute advantages in safety and environmental protection, and is more favored by researchers. However, wet processing has seasonal requirements for konjac, and more importantly, has a high cost, which limits its application in the industrial processing of konjac flour [29,30].

## 3. Chemical Structure and Physicochemical Properties of KGM

### 3.1. Chemical Structure of KGM

**Figure 3:**
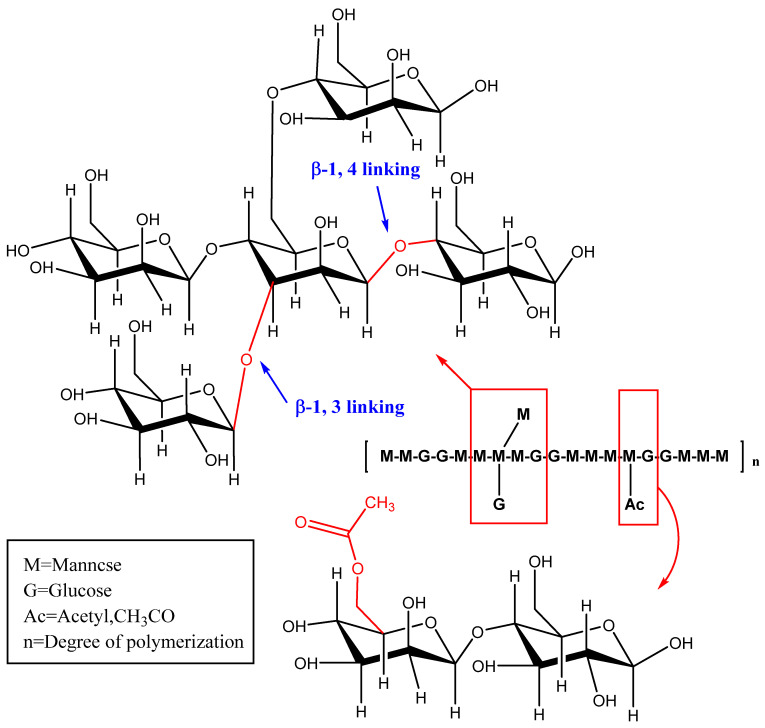
Schematic diagram of KGM structure.

The chemical structure of polysaccharides has a strong influence on their functional and nutritional properties or biological activity [31]. The basic framework of KGM is composed of D-glucose residues and D-mannose residues (molar ratio 1:1.6~1.7) and is polymerized by β-1,4-pyranoside bonds [32]. Part of the side chain can be formed at the C-3 position of the main chain mannose or the C-6 position of the sugar unit, by the linkage between acetyl groups (Figure 3) [33]. KGM has two native conformations, alpha (amorphous) and beta (crystalline) [34]. The molecular weight distribution of KGM is approximately normal since it is a relatively homogeneous polysaccharide. However, KGM has different molecular weight distributions that are affected by their origin, processing, and storage times [24]. It is reported that KGM has a mean molecular weight of 5.83 × 10^5^ g/mol [35].

### 3.2. Physical and Chemical Properties and Function of KGM in Food and Diabetes

**Figure 4:**
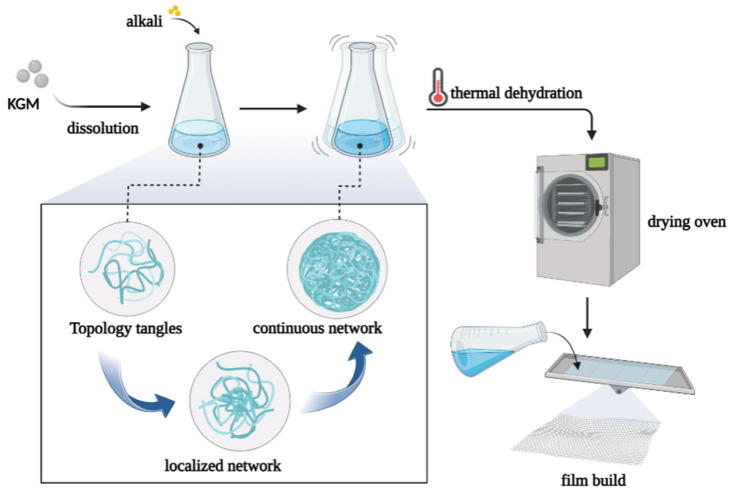
Alkali treatment causes the molecules of KGM to gather and form gels, which are then heated and dehydrated to obtain thin films.

When KGM is dissolved in water, it can maintain high performance in an acidic environment, but it is easily unstable in an alkaline environment. For example, these gels remain essentially unchanged in strength, even after repeated heating at 100 °C [36]. A possible explanation for this may be that the acetyl group of KGM is lost under alkaline conditions, which causes aggregation and entanglement of split self-body substrates, which eventually results in the formation of a localized, continuous gel reticular structure (Figure 4) [37]. These properties may also explain the reduced diffusion of glucose in the gut, such as the complex network of gel films that prevent glucose transfer through dialysis.

Furthermore, the role of KGM is also related to viscosity and rheological properties. Firstly, KGM can form a high viscosity solution because of its high molecular weight, strong adsorption to water, and electrical neutrality [31]. The apparent viscosity of 1% (*w*/*w*) KGM is about 30,000 cps, making it the most viscosity natural polysaccharide [38,39]. A moderate increase in dietary viscosity by KGM will help T2DM to meet the challenge of hunger in weight management. Secondly, fiber can lessen the rise in postprandial blood glucose and insulin concentration in both normal and diabetic individuals due to its rheological properties [40]. KGM solution is a typical pseudoplastic fluid with shear-thinning properties. The rheological curve can be fitted by the power law equation τ = KD^n^, where τ is shear stress (Pa), K is viscosity index (Pa·s^n^), and D is shear rate (s^−1^) [41]. The mechanism by which KGM improves metabolic control may be related to its rheological properties, such as increasing the viscosity of digestive juices and slowing down the absorption of food in the small intestine, thus reducing postprandial blood glucose and prolonging satiety.

The physical and chemical properties of KGM not only bring many benefits to diabetics, but they also provide more choices for the diabetic diet. Many konjac foods can be produced by using the gelling property of KGM, including konjac powder, konjac knot, konjac cake, konjac tofu, and konjac jelly. It not only meets people’s psychological and taste needs, but also maintains the effects of konjac weight loss and fullness, promotes oral health, enhances the growth and vitality of beneficial organisms in the colon, and has a combination of nutrients (such as cholesterol). KGM can also be used as a preservative and fat substitute. The diet of diabetics is affected by diseases, so they should reasonably distribute their food intake among three meals a day, KGM can be added to meat products as a fat substitute, which can effectively improve meat texture, thicken, reduce fat, and improve water retention [42]. Low-glycemic index (GI) diets are thought to reduce postprandial glycemia, resulting in more stable blood glucose concentrations [43,44]. For example, the GI of most fruits and vegetables is relatively low, so it is suitable for consumption by those with T2DM [45]. KGM can be used as a fresh-keeping agent for fruits and vegetables to give patients a better experience in vision and taste [46,47].

## 4. Biomedical Function of KGM to T2DM

**Figure 5:**
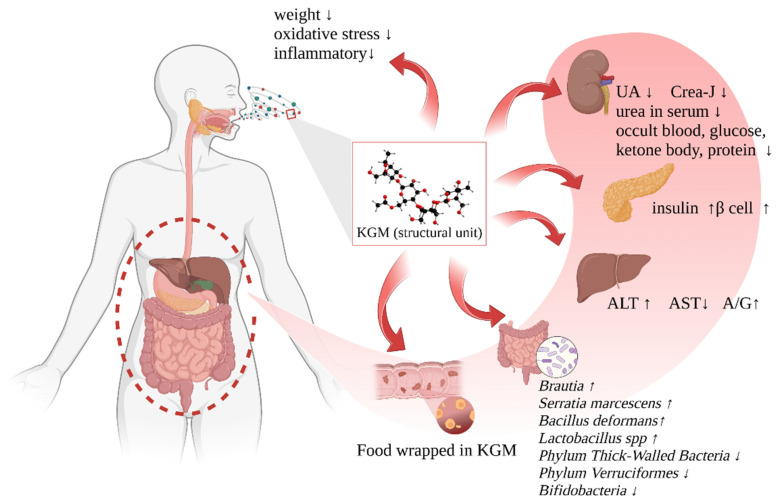
The benefits of the intake of KGM to the human body.

As a kind of natural polysaccharide with unique structure and physicochemical properties, KGM has shown brilliant medical value and prospects. Pharmacological studies on KGM have shown that it has the potential to regulate blood lipids, blood sugar, inflammation, oxidative stress, and intestinal microorganisms (Table 1) [48]. It can relieve the deterioration of T2DM by regulating physiological and biochemical indexes of various organs (Figure 5).

### 4.1. Regulate Blood Lipid

Dyslipidemia often occurs in the latent and developmental stages of T2DM and is the major cause of complications of diabetics. Therefore, the regulation of blood lipids is one of the therapeutic methods for T2DM [49,50]. Plenty of studies have observed that KGM regulates the metabolic parameters of lipid and cholesterol levels [51]. The long-term consumption of low-dose KGM can improve LDL-C and cholesterol, and reduce blood lipid levels [51]. According to clinical studies, diabetic patients with high cholesterol levels can reduce their cholesterol levels by adding 0.7 g of KGM per 100 calories to their diet [52]. Although the mechanism of the hypolipidemic effect has not been fully understood, it is presumed to be achieved by increasing the excretion of sterols or bile acids, and this presumption has been confirmed by experimental studies in animals [53]. Compared with other dietary supplements, KGM lowers the primary endpoint of metabolic control and various indicators of diabetes such as plasma cholesterol, LDL-C, total/HDL-C ratio, and ApoB [22]. KGM supplementation increased the expression of lipid metabolism-related genes (PPARα, CPT1, Hs1) and lipid transport-related genes (FABP1, apoB100, and CD36), and decreased the expression of lipid synthesis-related genes (srebp1, PPARγ, Fas) [54]. It is apparent that KGM is capable of improving the weight and blood lipids of most obese people, and has the potential to prevent and improve obesity in a limited sense.

It can improve obesity through the following mechanisms: (I) KGM is difficult to be decomposed by human enzymes (mainly endo-1,4-beta-mannanase) [17]; (II) KGM is a soluble fiber that forms a viscous, gel-like mass when hydrated in the stomach, which contributes to a reduction in the rate of gastric emptying and can induce satiety [55]; (III) KGM promotes intestinal peristalsis [56]. (IV) KGM metabolizes energy by excreting feces; (V) the hydrolysate of KGM can stimulate the growth of lactic acid bacteria and play a probiotic role [57]. KGM can also be used in combination with drugs, which is quite effective in the treatment of obese teenagers [58]. Orlistat has become the only drug approved for the treatment of adolescent obesity in the European Union. KGM can reduce the side effects of orlistat, such as transient gastrointestinal adverse reactions [58].

### 4.2. Maintenance of Glucose Homeostasis

**Figure 6:**
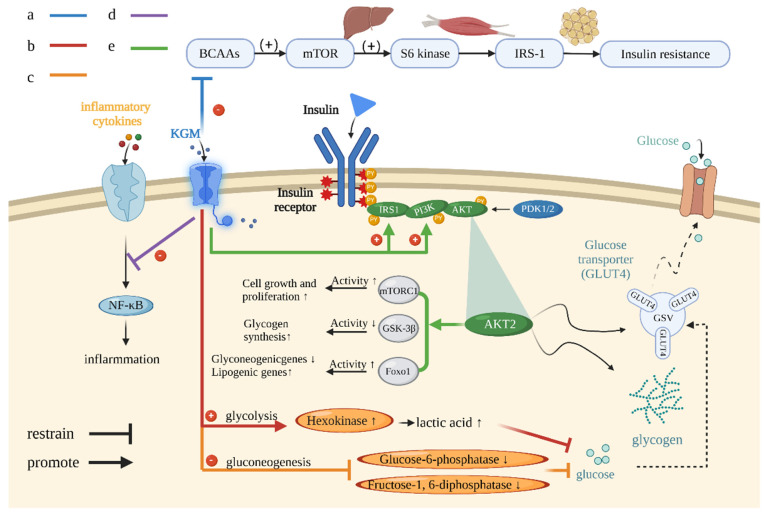
The related mechanism of maintaining sugar homeostasis by KGM. (**a**) KGM reduces insulin resistance through the BCAA pathway. (**b**) KGM increases hexokinase activity and glycolysis. (**c**) KGM regulates the activity of fructose-1 and glucose-6-bisphosphatase and reduces gluconeogenesis. (**d**) KGM inhibits the signal pathway of related inflammatory factors and reduces the immune inflammatory response associated with diabetes. (**e**) KGM up-regulates the expression of IRS1 and PI3K to improve the insulin signaling pathway.

The uptake, utilization, storage, regeneration, and metabolism of glucose are important for maintaining glucose homeostasis in patients with diabetes. An imbalance in glucose homeostasis occurs for individuals with T2DM [59]. Insulin is the most important component of blood sugar control. Therefore, impaired signal transmission will give rise to a disturbance of blood sugar levels [60]. It was found that insulin signaling genes and proteins reduced in diabetic rats, and KGM consumption could increase gene up-regulation and insulin pathway expression, resulting in normalized insulin secretion and lowered blood sugar levels [61]. In addition, insulin receptor substrate 1 (IRS1) and phosphatidylinositol 3-kinase (PI3K) play crucial roles in mediating insulin metabolism. Reduced IRS1 activity can lead to mutations in the tyrosine kinase hinsr gene in diabetics, so as to disrupt insulin signaling [62]. An experiment found that KGM can reduce the mice’s body weight and blood sugar level significantly, restore the normal proliferation of pancreatic β-cells, and increase IRS1 and PI3K expression levels (Figure 6e) [63]. This suggests that the reasons why KGM can prevent insulin resistance in diabetic rats may be due to the following two points: (I) inducing the expression of activating proteins related to pancreatic β-cell repair and (II) regeneration of pancreatic β-cells through receptor tyrosine kinase pathway.

Reducing glucose intake is one of the treatments to lower postprandial glucose in patients with T2DM [64]. There are two main mechanisms associated with this therapeutic target. The first is the reduction of the diffusion rate of glucose. This is related to the ability of different molecules to thicken and swell the hydration network and the subsequent size of the resulting viscosity [65]. The second mechanism is the inhibition of carbohydrate hydrolases (α-amylase and α-glucosidase) in the small intestine. KGM exerts its hypoglycemic effect mainly through the first modality. The dissolved KGM is in the state of gel, which can wrap nutrients, slow down the flow of food in the digestive tract, prolong the residence time of food paste in the gastric cavity, form a protective membrane barrier, and effectively inhibit the value of postprandial blood glucose. Although there is no direct evidence that KGM has the second mechanism of regulation, there is evidence that konjac can lower blood glucose by inhibiting α-amylase and α-glucosidase activity [66].

The increase in circulating blood glucose concentration plays a key role in the pathogenesis of chronic diabetic complications [67,68]. The combination of high sugar and high fat will often lead to a series of human health problems, including eye, kidney, heart, liver, nerve, and other dysfunctions. One element influencing the onset and progression of diabetic nephropathy is a long-term aberrant rise in glomerular microcirculatory filtration pressure. Animal experiments have shown that KGM can treat diabetic nephropathy, normalize rat glomerular structure, lower serum uric acid, creatinine-J, urine occult blood, blood glucose, a ketone body, and protein levels [69]. The ability of the liver to store glycogen is an important indicator of T2DM. KGM can promote the synthesis of glycogen in the liver, reducing blood sugar and alleviating the progression of diabetes, which is beneficial to the protection of the liver. Elevated serum alanine aminotransferase (ALT) and aspartate aminotransferase (AST) reflect the degree of hepatocellular damage, and serum alkaline phosphatase (ALP) activity represents the severity of cholestasis [70]. An elevated ALT level is closely related to T2DM, causing abnormal metabolism of leucine, lysine, and glutamic acid. A study was conducted on mice with liver injury prepared by subcutaneous injection of CCl_4_ as a model [71], it was proved that the administration of KGM could down-regulate the abnormal increase of ALT and AST in mice serum, up-regulate the A/G ratio in serum, and increase the abnormally low superoxide dismutase (SOD) content and decrease the abnormally high malondialdehyde content in the liver homogenate of mice caused by liver injury. This demonstrates the protective effect of KGM on experimental liver injury caused by CCl_4_ in mice.

In glucose metabolism, KGM greatly affects the enzymatic activity of glycolytic and gluconeogenic enzymes. As a rate-limiting enzyme of glycolysis, hexokinase regulates glucose uptake and energy production by converting glucose into glucose 6-phosphate. When blood glucose levels fall below normal requirements, glucose-6-phosphate acts as a gluconeogenesis enzyme in the liver, releasing glucose through glucose-6-phosphatase [72]. Researchers found that in diabetic rats, hexokinase, glucose-6-phosphate dehydrogenase, and glycogen levels were significantly reduced (*p* < 0.05), while gluconeogenesis (glucose-6-phosphatase) was significantly increased. In diabetic rats administered 80 mg/kg of KGM, the levels of hexokinase, glucose-6-phosphate dehydrogenase, and glycogen were increased, but the levels of gluconeogenesis enzymes were decreased [73,74]. The intensity of gluconeogenic enzymes was significantly reduced, which led to the hypothesis that KGM treatment could improve the activity of enzymes involved in glucose metabolism to improve insulin sensitivity and up-regulate more glucose for energy production (Figure 6b,c) [61].

### 4.3. Regulation of Oxidative Stress and Inflammation

As a systemic chronic disease, diabetes is bound to be affected by oxidative stress and inflammation [75,76]. Some inflammatory markers such as TNF-α, IL-6, and monocyte chemoattractant protein-1 (MCP-1) have been shown to be associated with adipose tissue and are the primary causes of insulin resistance [77,78]. Detecting and regulating changes in oxidative stress and inflammation may prevent the progression of diabetes. Studies have found that KGM can increase aromatic amino acid (AAA) metabolism-related intestinal bacteria (*Lactobacillus*, *Ruminococcus-1*, and *Bifidobacterium*) and AAA metabolites (kynurenic acid, desaminotyrosine, 3-hydroxy-3-(3-hydroxyphenyl) propanoic acid-O-sulphate and hippuric acid), which may also contribute to the reduction of systemic inflammation and thus reduce symptoms of oxidative stress and T2DM [79]. KGM appears to regulate oxidative stress and inflammation positively, which indicates that it may be effective in preventing or treating diabetes, although further research and data are necessary to confirm this.

### 4.4. Effects on Gut Microbes

The gut microbiota play a crucial role in the body’s physiological processes [15,80,81]. Intestinal microflora can regulate the Mammalian target rapamycin (mTORC) signal pathway, thus inhibit tyrosine phosphorylation, reduce protein level, down-regulate insulin signal transduction, and exerting the effect of intestinal microorganisms on diabetes [82]. Diabetic patients have different intestinal flora species as well as different numbers than healthy individuals due to metabolic dysregulation. The levels of Enterococci in patients with diabetes will increase, while those of *Bifidobacterium vulgatus* and other *Bifidobacterium* species decrease when their blood glucose rises, leading to the aggravation of diabetes. Conditional pathogenic bacteria, such as *Bacteroides*, *Escherichia coli*, and *Desulfovibrio*, have been observed to increase in diabetic patients [83]. Meanwhile, most of the metabolites and products of these microflorae are mediated by mTOR pathway [84]. For example, BCAA is synthesized by intestinal microorganisms. The high content of BCAA can activate mTOR and its downstream effector S6K1 in the liver, muscle, and adipose tissue, and sustained activation leads to serine phosphorylation of IRS-1, which inhibits IRS-1 and leads to insulin resistance (Figure 6a) [85]; mTORC activates anabolism (protein and fat biosynthesis) and inhibits autophagy. Its overactivation may lead to diabetes [84]. The reason why KGM reduces insulin resistance may be achieved by down-regulating the abundance of BCAA-synthesizing-associated flora [86], including *Clostridium* spp., *Bacteroides* spp., *Prevotella* spp., *Klebsiella* spp., *E. coli*, *Streptococcus* spp., and *S. aureus* [86].

**Table 1:** Summary of biomedical functions of diabetes mellitus by KGM.

KGM Activity	Model	Study Design and Period (DUS)	Dosage Form (DSF)	Results	Reference
Regulation of blood lipids	Eight in adults and four in children	3 g/day; three weeks	Unlimited	Reduced LDL cholesterol and non-HDL cholesterol of 10% and 7%.	[51]
	Twenty-two diabetic subjects	3.6 g/day; 28 days	Gelatin capsules	Alleviated the elevated cholesterol, LDL-cholesterol, apo B, and ratios of total/HDL-cholesterol.	[52]
	Seven hundred and twenty healthy fish	With 0, 0.5, 1 and 2% KGM twice a day; 60 days	Fodder	Improved growth performance, antioxidant activity, immune response, and lipid metabolism in juvenile pompano.	[54]
Regulation of blood sugar	Six rats in each group	I-control rats fed standard pellet diet alone II-KGM control (120 mg/kg body weight (b.w.)) III-T2DM (HFD + STZ-40 mg/kg b.w.) IV-T2DM + KGM (80 mg/kg b.w.) V-T2DM + RSG (4 mg/kg b.w.); 28 days	Fodder	Improved cell glucose-tolerance; regulated glycolytic, gluconeogenesis enzymes; reduced the stored glycogen in the liver, and restored liver enzymes.	[61]
	Three-month-old male	0.06 g/mg/kg b.w. and 0.12 g/mg/kg b.w.; one month	Fodder	Significantly increased IRS-1 level expression, proliferated properly and consistently increased the PI3-K expression level.	[63]
	Twelve-week-old male Wistar rats (*n* = 8)	102 mg/kg b.w.; at0,30,60, and 120 min	Konjac solution	Konjac has hypoglycemic and antioxidant activities in vitro and in vivo.	[66]
	Seven-week-old male Wistar rats (180–220 g)	160 mg/kg b.w. of glucomannans; treatment for once every day; gavage for 4 weeks	Fodder	Lower levels of fasting blood glucose, serum insulin, and glycated serum protein; improve urea cycle, metabolism of lipid, glucose, and amino acids.	[69]
	Rats (weight 22~28 g)	50,100,200 mg/kg b.w.; once a day; six days	Konjac solution	The levels of ALT and AST decreased, the ratio of serum albumin to globulin increased, and hepatocytes were protected.	[71]
Regulation of oxidative stress and inflammation	Male C57BL/6J mice (body weight 20 ± 2 g)	500 mg/kg b.w.; once a day; 4 weeks	Konjac solution	Lactobacillus, Ruminococcus_1 and Bifidobacterium and AAA metabolites such as kynurenic acid, desaminotyrosine, 3hydroxy-3-(3hydroxyphenyl) propanoic acid-O-sulphate, and hippuric acid were added.	[79]
Effects on gut microbes	Wistar rats (180–220 g)	2 mL/200 g of BW; once daily for 4 weeks	fodder	Decreased the abundance of microbial BCAA biosynthesis-related genes and ameliorated the host BCAA metabolism.	[86]

## 5. Conclusions and Future Prospect

**Figure 7:**
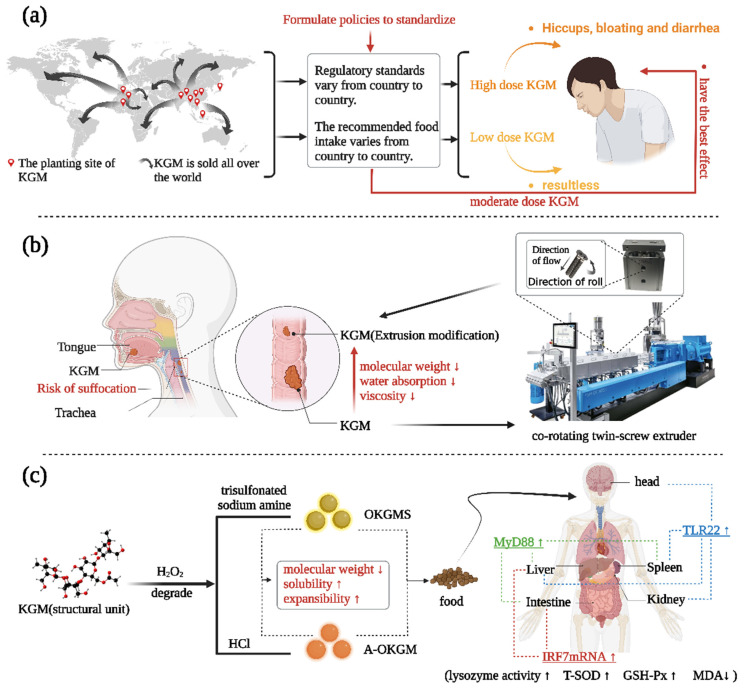
Some solutions to KGM’s problems. (**a**) The establishment of a unified regulatory system can better regulate the average daily intake of KGM and reduce the side effects. (**b**) KGM that has undergone physical modification can lessen the asphyxia risk. (**c**) Chemically altered KGM derivatives can improve the body’s immunological indicators.

There are several advantages of KGM and there are also some problems. First, high doses cause minor side effects with high doses, such as hiccups, bloating, and diarrhea [87,88]. Fortunately, there have only been a handful of reports on side effects associated with KGM in a normal diet. It has been demonstrated that a short-term intake of 3.9 g of KGM per day does not cause changes in bowel function [89]. Currently, there is no unified regulatory standard on a global scale within KGM, which needs to rely on the improvement and perfection of the food management industry as it advances. The KGM also provides an opportunity to formulate or revise FSMP-related policies to promote the rapid and orderly development of the FSMP industry (Figure 7a) [90,91,92].

Secondly, the highwater absorption of KGM is associated with the risk of asphyxiation. Viscosity plays a crucial role in the swallowing process [93]. The thicker liquid gives the muscles more time to react by slowing down the flow of food in the mouth and throat stages [82], and the likelihood of swallowing incidents is decreased as a result [94]. After, KGM repeated machine extrusion and high temperature grinding the water absorption and viscosity will significantly reduce, which can reduce the risk of asphyxiation of products containing KGM (Figure 7b) [95].

Thirdly, the solubility of natural KGM in water decreases with increasing molecular weight, and it has several shortcomings as a feed additive, such as low solubility, high expansibility, and ease to cause abdominal distension. The molecular weight of KGM degraded by H_2_O_2_ is significantly lower than that of raw materials, and it has peculiar solubility and does not easily cause abdominal distension. In addition, studies have shown that when the molecular weight of polysaccharides decreases to 100–200 kDa, the biological activity will be significantly increased [96]. After *S. prenanti* ate feed containing oxidized konjac glucomannan sulfates (OKGMS) and acidolysis-oxidized konjac glucomannan (A-OKGM), its growth and immune function were improved (Figure 7c) [97,98].

Fourthly, whiteness is one of the important indicators to measure the quality of konjac and its products, and the market value of konjac powder will decrease after browning. Therefore, in the initial processing of konjac, SO_2_ fumigation or sulfite soaking is usually used to protect the color of konjac powder, which may lead to sulfur dioxide residue in KGM [25]. At present, it can be reduced by improving drying methods (vacuum freeze drying, microwave drying) or using natural browning inhibitors (such as citric acid, mercaptan, ascorbic acid, and oxalic acid) [99].

As the challenges faced by KGM in production applications are gradually overcome, its demonstrated advantages as a biomedical function of FSMP are attracting attention. KGM can improve the taste, flavor, and visual enjoyment of food as an additive. It can also play a powerful role as a dietary supplement and in combination with drugs [100]. Moreover, the hypoglycemic effect of different purities of KGM is also different, and one study found that 90% of the hypoglycemic effect is the best [101,102,103,104]. Thus, KGM may also replace the traditional sugars in drugs, bringing a blessing to patients with obesity, hyperglycemia, hypertension, and metabolic syndrome. The integration of such dietary fiber into conventional medical practice may reduce the required drug dose, thus improving the overall economic benefits and therapeutic efficacy [39]. Furthermore, continuous innovation of product dosage forms, continuous refinement of disease coverage, gradual precision of nutritional interventions, and accelerated exploration of clinical treatment concepts will open up industrial development paths for FSMP based on KGM.

## Data Availability

Not applicable.

## Abbreviations

T2DM	type 2 diabetes mellitus
KGM	Konjac glucomannan
FSMP	food for special medical purpose
GI	glycemic index
IRS1	insulin receptor substrate 1
PI3K	phosphatidylinositol 3-kinase
ALT	alanine aminotransferase
AST	aspartate aminotransferase
ALP	serum alkaline phosphatase
SOD	superoxide dismutase
OKGM	oxidized konjac glucomannan sulfates
A-OKGM	acidolysis-oxidized konjac glucomannan
BCAA	Branched Chain Amino Acid

## References

[1] Fletcher B., Gulanick M., Lamendola C. (2002). Risk Factors for Type 2 Diabetes Mellitus. J. Cardiovasc. Nurs..

[2] American Diabetes Association (2013). Diagnosis and Classification of Diabetes Mellitus. Diabetes Care.

[3] Griffin S. (2022). Diabetes precision medicine: Plenty of potential, pitfalls and perils but not yet ready for prime time. Diabetologia.

[4] Maskarinec G., Raquinio P., Kristal B., Wilkens L., Franke A., Lim U., Marchand L.L., Lampe J., Hullar M. (2020). The Gut Microbiome and Diabetes Status in the Multiethnic Cohort. Curr. Dev. Nutr..

[5] Jiang H., Cai M., Shen B., Wang Q., Zhang T., Zhou X. (2022). Synbiotics and Gut Microbiota: New Perspectives in the Treatment of Type 2 Diabetes Mellitus. Foods.

[6] Sun J., Ren J., Hu X., Hou Y., Yang Y. (2021). Therapeutic effects of Chinese herbal medicines and their extracts on diabetes. Biomed. Pharmacother..

[7] Chen L., Magliano D.J., Zimmet P.Z. (2012). The worldwide epidemiology of type 2 diabetes mellitus—Present and future perspectives. Nat. Rev. Endocrinol..

[8] Wu Y., Ding Y., Tanaka Y., Zhang W. (2014). Risk Factors Contributing to Type 2 Diabetes and Recent Advances in the Treatment and Prevention. Int. J. Med. Sci..

[9] Zhou K., Pedersen H.K., Dawed A.Y., Pearson E.R. (2016). Pharmacogenomics in diabetes mellitus: Insights into drug action and drug discovery. Nat. Rev. Endocrinol..

[10] Lee S.-H., Yoon K.-H. (2021). A Century of Progress in Diabetes Care with Insulin: A History of Innovations and Foundation for the Future. Diabetes Metab. J..

[11] Ojo O. (2019). Dietary Intake and Type 2 Diabetes. Nutrients.

[12] Mao T., Huang F., Zhu X., Wei D., Chen L. (2021). Effects of dietary fiber on glycemic control and insulin sensitivity in patients with type 2 diabetes: A systematic review and meta-analysis. J. Funct. Foods.

[13] Connolly M.L., Lovegrove J.A., Tuohy K.M. (2010). Konjac glucomannan hydrolysate beneficially modulates bacterial composition and activity within the faecal microbiota. J. Funct. Foods.

[14] Tester R.F., Al-Ghazzewi F.H. (2016). Beneficial health characteristics of native and hydrolysed konjac (*Amorphophallus konjac*) glucomannan: Health characteristics of native and hydrolysed konjac glucomannan. J. Sci. Food Agric..

[15] Wu X., Song M., Qiu P., Li F., Wang M., Zheng J., Wang Q., Xu F., Xiao H. (2018). A metabolite of nobiletin, 4′-demethylnobiletin and atorvastatin synergistically inhibits human colon cancer cell growth by inducing G0/G1 cell cycle arrest and apoptosis. Food Funct..

[16] Zhou Y., Qin J., Wang Y., Wang Y., Cheng Y. (2019). Gastrointestinal and metabolic effects of noodles-based konjac glucomannan in rats. Food Nutr. Res..

[17] Nagasawa T., Kimura T., Yoshida A., Tsunekawa K., Araki O., Ushiki K., Ishigaki H., Shoho Y., Suda I., Hiramoto S. (2021). Konjac Glucomannan Attenuated Triglyceride Metabolism during Rice Gruel Tolerance Test. Nutrients.

[18] Yoshida A., Kimura T., Tsunekawa K., Araki O., Ushiki K., Ishigaki H., Shoho Y., Suda I., Hiramoto S., Murakami M. (2020). Glucomannan Inhibits Rice Gruel-Induced Increases in Plasma Glucose and Insulin Levels. Ann. Nutr. Metab..

[19] Adams J., Hofman K., Moubarac J.-C., Thow A.M. (2020). Public health response to ultra-processed food and drinks. BMJ.

[20] Gibney M.J., Forde C.G., Mullally D., Gibney E.R. (2017). Ultra-processed foods in human health: A critical appraisal. Am. J. Clin. Nutr..

[21] Monteiro C.A., Cannon G., Levy R.B., Moubarac J.-C., Louzada M.L., Rauber F., Khandpur N., Cediel G., Neri D., Martinez-Steele E. (2019). Ultra-processed foods: What they are and how to identify them. Public Health Nutr..

[22] Devaraj R.D., Reddy C.K., Xu B. (2019). Health-promoting effects of konjac glucomannan and its practical applications: A critical review. Int. J. Biol. Macromol..

[23] Parry J.-M., Imeson A. (2009). Konjac Glucomannan. Food Stabilisers, Thickeners and Gelling Agents.

[24] Khan H. (2019). Marya Konjac (*Amorphophallus konjac*). Nonvitamin and Nonmineral Nutritional Supplements.

[25] Ye S., Zongo A.W.-S., Shah B.R., Li J., Li B. (2021). Konjac Glucomannan (KGM), Deacetylated KGM (Da-KGM), and Degraded KGM Derivatives: A Special Focus on Colloidal Nutrition. J. Agric. Food Chem..

[26] Shi Y., Tao Y., Lu Y., Fei X. (1998). Morphology of starch and manna granules in corms of Amorphophallus COn/ac. Guihaia.

[27] NIE C., GAO Q. (2022). Research Progress on Deep Processing and Application of Konjak. Food Sci. Technol..

[28] Xu W., Wang S., Ye T., Jin W., Liu J., Lei J., Li B., Wang C. (2014). A simple and feasible approach to purify konjac glucomannan from konjac flour—Temperature effect. Food Chem..

[29] Ye T., Wang L., Xu W., Liu J., Wang Y., Zhu K., Wang S., Li B., Wang C. (2014). An approach for prominent enhancement of the quality of konjac flour: Dimethyl sulfoxide as medium. Carbohydr. Polym..

[30] Yuan Z., Wu D., Wu H., Li X. (2003). China journal of Chinese Materia. Medica.

[31] Tester R., Al-Ghazzewi F. (2017). Glucomannans and nutrition. Food Hydrocoll..

[32] Cui T., Liu R., Wu T., Sui W., Zhang M. (2019). Influence of Konjac Glucomannan and Frozen Storage on Rheological and Tensile Properties of Frozen Dough. Polymers.

[33] Jiang M., Li H., Shi J., Xu Z. (2018). Depolymerized konjac glucomannan: Preparation and application in health care. J. Zhejiang Univ. Sci. B.

[34] Meng F., Liu D., LI Y. (2016). Research progress of the structures, properties and modifications of Konjacglucomannan. Sci. Technol. Food Ind..

[35] Katsuraya K., Okuyama K., Hatanaka K., Oshima R., Sato T., Matsuzaki K. (2003). Constitution of konjac glucomannan: Chemical analysis and 13C NMR spectroscopy. Carbohydr. Polym..

[36] Yang D., Yuan Y., Wang L., Wang X., Mu R., Pang J., Xiao J., Zheng Y. (2017). A Review on Konjac Glucomannan Gels: Microstructure and Application. Int. J. Mol. Sci..

[37] Hou Y., Nie J. (2022). Shaoping Study and application on the deacetylated gelation of konjac glucomannan. J. Shaanxi Norm. Univ..

[38] Du X., Li J., Chen J., Li B. (2012). Effect of degree of deacetylation on physicochemical and gelation properties of konjac glucomannan. Food Res. Int..

[39] Chua M., Baldwin T.C., Hocking T.J., Chan K. (2010). Traditional uses and potential health benefits of Amorphophallus konjac K. Koch ex N.E.Br. J. Ethnopharmacol..

[40] InterAct Consortium (2015). Dietary fibre and incidence of type 2 diabetes in eight European countries: The EPIC-InterAct Study and a meta-analysis of prospective studies. Diabetologia.

[41] Yoshimura M., Nishinari K. (1999). Dynamic viscoelastic study on the gelation of konjac glucomannan with different molecular weights. Food Hydrocoll..

[42] Chen J., Zhao J., Li X., Liu Q., Kong B. (2021). Composite Gel Fabricated with Konjac Glucomannan and Carrageenan Could Be Used as a Cube Fat Substitute to Partially Replace Pork Fat in Harbin Dry Sausages. Foods.

[43] Vlachos D., Malisova S., Lindberg F.A., Karaniki G. (2020). Glycemic Index (GI) or Glycemic Load (GL) and Dietary Interventions for Optimizing Postprandial Hyperglycemia in Patients with T2 Diabetes: A Review. Nutrients.

[44] Zafar M.I., Mills K.E., Zheng J., Regmi A., Hu S.Q., Gou L., Chen L.-L. (2019). Low-glycemic index diets as an intervention for diabetes: A systematic review and meta-analysis. Am. J. Clin. Nutr..

[45] Atkinson F.S., Brand-Miller J.C., Foster-Powell K., Buyken A.E., Goletzke J. (2021). International tables of glycemic index and glycemic load values 2021: A systematic review. Am. J. Clin. Nutr..

[46] Li X., Xiao N., Xiao G., Bai W., Zhang X., Zhao W. (2021). Lemon essential oil/vermiculite encapsulated in electrospun konjac glucomannan-grafted-poly (acrylic acid)/polyvinyl alcohol bacteriostatic pad: Sustained control release and its application in food preservation. Food Chem..

[47] Hashemi S.M.B., Jafarpour D. (2020). The efficacy of edible film from Konjac glucomannan and saffron petal extract to improve shelf life of fresh-cut cucumber. Food Sci. Nutr..

[48] Zhao Y., Jayachandran M., Xu B. (2020). In vivo antioxidant and anti-inflammatory effects of soluble dietary fiber Konjac glucomannan in type-2 diabetic rats. Int. J. Biol. Macromol..

[49] Zhang C., Chen J., Yang F. (2014). Konjac Glucomannan, a Promising Polysaccharide for OCDDS. Carbohydr. Polym..

[50] Lu Y., Zhang J., Zhang Z., Liang X., Liu T., Yi H., Gong P., Wang L., Yang W., Zhang X. (2021). Konjac Glucomannan with Probiotics Acts as a Combination Laxative to Relieve Constipation in Mice by Increasing Short-Chain Fatty Acid Metabolism and 5-Hydroxytryptamine Hormone Release. Nutrition.

[51] Hayeeawaema F., Wichienchot S., Khuituan P. (2020). Amelioration of Gut Dysbiosis and Gastrointestinal Motility by Konjac Oligo-Glucomannan on Loperamide-Induced Constipation in Mice. Nutrition.

[52] Patel J. (2008). Diabetes: Managing dyslipidaemia. BMJ Clin. Evid..

[53] Szalat A., Durst R., Leitersdorf E. (2016). Managing dyslipidaemia in type 2 diabetes mellitus. Best Pract. Res. Clin. Endocrinol. Metab..

[54] Ho H.V.T., Jovanovski E., Zurbau A., Blanco Mejia S., Sievenpiper J.L., Au-Yeung F., Jenkins A.L., Duvnjak L., Leiter L., Vuksan V. (2017). A systematic review and meta-analysis of randomized controlled trials of the effect of konjac glucomannan, a viscous soluble fiber, on LDL cholesterol and the new lipid targets non-HDL cholesterol and apolipoprotein B. Am. J. Clin. Nutr..

[55] Chen H.-L., Sheu W.H.-H., Tai T.-S., Liaw Y.-P., Chen Y.-C. (2003). Konjac Supplement Alleviated Hypercholesterolemia and Hyperglycemia in Type 2 Diabetic Subjects—A Randomized Double-Blind Trial. J. Am. Coll. Nutr..

[56] Dziadek K., Kopeć A., Piątkowska E., Leszczyńska T. (2019). High-Fructose Diet-Induced Metabolic Disorders Were Counteracted by the Intake of Fruit and Leaves of Sweet Cherry in Wistar Rats. Nutrients.

[57] Li Y., Liang S., Shao Y., Li Y., Chen C., You C., Monroig Ó., Rahimnejad S., Tocher D.R., Wang S. (2021). Impacts of dietary konjac glucomannan supplementation on growth, antioxidant capacity, hepatic lipid metabolism and inflammatory response in golden pompano (*Trachinotus ovatus*) fed a high fat diet. Aquaculture.

[58] Keleszade E., Willner T., Patterson M., Trangmar S., Kolida S., Costabile A. (2020). A pilot study to assess the effect of a fibre and mineral formulation on satiety and satiation when taken as part of a calorie restriction diet in overweight and obese women. J. Funct. Foods.

[59] Guo L., Yokoyama W., Chen M., Zhong F. (2021). Konjac glucomannan molecular and rheological properties that delay gastric emptying and improve the regulation of appetite. Food Hydrocoll..

[60] Al-Ghazzewi F.H., Tester R.F. (2012). Efficacy of cellulase and mannanase hydrolysates of konjac glucomannan to promote the growth of lactic acid bacteria. J. Sci. Food Agric..

[61] Rogovik A.L., Goldman R.D. (2009). Should weight-loss supplements be used for pediatric obesity?. Can. Fam. Physician.

[62] Röder P.V., Wu B., Liu Y., Han W. (2016). Pancreatic regulation of glucose homeostasis. Exp. Mol. Med..

[63] Bettedi L., Yan A., Schuster E., Alic N., Foukas L.C. (2020). Increased mitochondrial and lipid metabolism is a conserved effect of Insulin/PI3K pathway downregulation in adipose tissue. Sci. Rep..

[64] Li X., Jayachandran M., Xu B. (2021). Antidiabetic effect of konjac glucomannan via insulin signaling pathway regulation in high-fat diet and streptozotocin-induced diabetic rats. Food Res. Int..

[65] Fatchiyah F., Christian N., Soeatmadji D. (2013). Reducing IRS-1 Activation Cause Mutation of Tyrosine Kinase Domain hINSR Gene on Type-2 Diabetes Mellitus Patients. Bioinformation.

[66] Fatchiyah F., Nurmasari D.A., Masruro N., Rohmah N.R., Triprisila L.F., Mulyati M., Yamada T., Ohta T. (2019). Level of mRNA Insulin Gene and Blood Glucose STZ-Induced Diabetic Rat are Improved by Glucomannan of Amorphophallus muelleri Blume from East Java Forest Indonesia. J. Trop. Life Sci..

[67] Rasouli H., Hosseini-Ghazvini S.M.-B., Adibi H., Khodarahmi R. (2017). Differential α-amylase/α-glucosidase inhibitory activities of plant-derived phenolic compounds: A virtual screening perspective for the treatment of obesity and diabetes. Food Funct..

[68] Fabek H., Messerschmidt S., Brulport V., Goff H.D. (2014). The effect of in vitro digestive processes on the viscosity of dietary fibres and their influence on glucose diffusion. Food Hydrocoll..

[69] Gamboa-Gómez C.I., Guerrero-Romero F., Sánchez-Meraz M.A., Simental-Mendía L.E. (2020). Hypoglycemic and antioxidant properties of konjac (*Amorphophallus konjac*) in vitro and in vivo. J. Food Biochem..

[70] Spanakis E.K., Singh L.G., Siddiqui T., Sorkin J.D., Notas G., Magee M.F., Fink J.C., Zhan M., Umpierrez G.E. (2020). Association of glucose variability at the last day of hospitalization with 30-day readmission in adults with diabetes. BMJ Open Diab. Res. Care.

[71] Tong L., Chi C., Zhang Z. (2018). Association of various glycemic variability indices and vascular outcomes in type-2 diabetes patients: A retrospective study. Medicine.

[72] Chen H., Nie Q., Hu J., Huang X., Zhang K., Nie S. (2019). Glucomannans Alleviated the Progression of Diabetic Kidney Disease by Improving Kidney Metabolic Disturbance. Mol. Nutr. Food Res..

[73] Iluz-Freundlich D., Zhang M., Uhanova J., Minuk G.Y. (2020). The relative expression of hepatocellular and cholestatic liver enzymes in adult patients with liver disease. Ann. Hepatol..

[74] Lin H.-M., Pang J., Deng S.-G. (2009). Protective Effect of Konjac Glucomannan on Carbon Tetrachloride Induced Liver Injury in Mice. J. Zhejiang Ocean. Univ..

[75] Tang B.L. (2020). Glucose, glycolysis, and neurodegenerative diseases. J. Cell. Physiol..

[76] Ahmed D., Khan M.I., Sharma M., Khan M.F. (2018). Novel pentacyclic triterpene isolated from seeds of *Euryale Ferox Salisb*. ameliorates diabetes in streptozotocin induced diabetic rats. Interdiscip. Toxicol..

[77] Subash-Babu P., Ignacimuthu S., Alshatwi A.A. (2015). Nymphayol increases glucose-stimulated insulin secretion by RIN-5F cells and GLUT4-mediated insulin sensitization in type 2 diabetic rat liver. Chem.-Biol. Interact..

[78] Wellen K.E. (2005). Inflammation, stress, and diabetes. J. Clin. Investig..

[79] Asmat U., Abad K., Ismail K. (2016). Diabetes mellitus and oxidative stress—A concise review. Saudi Pharm. J..

[80] Anto Michel N., Colberg C., Buscher K., Sommer B., Pramod A.B., Ehinger E., Dufner B., Hoppe N., Pfeiffer K., Marchini T. (2018). Inflammatory Pathways Regulated by Tumor Necrosis Receptor–Associated Factor 1 Protect From Metabolic Consequences in Diet-Induced Obesity. Circ. Res..

[81] Bourebaba L., Bedjou F., Röcken M., Marycz K. (2019). Nortropane alkaloids as pharmacological chaperones in the rescue of equine adipose-derived mesenchymal stromal stem cells affected by metabolic syndrome through mitochondrial potentiation, endoplasmic reticulum stress mitigation and insulin resistance alleviation. Stem. Cell Res. Ther..

[82] Jeon J., Jang J., Park K. (2018). Effects of Consuming Calcium-Rich Foods on the Incidence of Type 2 Diabetes Mellitus. Nutrients.

[83] Song S., Lee J.E. (2018). Dietary Patterns Related to Triglyceride and High-Density Lipoprotein Cholesterol and the Incidence of Type 2 Diabetes in Korean Men and Women. Nutrients.

[84] Chen H., Ji H., Kong X., Lei P., Yang Q., Wu W., Jin L., Sun D. (2021). Bacterial Ghosts-Based Vaccine and Drug Delivery Systems. Pharmaceutics.

[85] Jung M.-J., Lee J., Shin N.-R., Kim M.-S., Hyun D.-W., Yun J.-H., Kim P.S., Whon T.W., Bae J.-W. (2016). Chronic Repression of mTOR Complex 2 Induces Changes in the Gut Microbiota of Diet-induced Obese Mice. Sci. Rep..

[86] Eckburg P.B., Bik E.M., Bernstein C.N., Purdom E., Dethlefsen L., Sargent M., Gill S.R., Nelson K.E., Relman D.A. (2005). Diversity of the Human Intestinal Microbial Flora. Science.

[87] Krebs M., Brunmair B., Brehm A., Artwohl M., Szendroedi J., Nowotny P., Roth E., Fürnsinn C., Promintzer M., Anderwald C. (2007). The Mammalian Target of Rapamycin Pathway Regulates Nutrient-Sensitive Glucose Uptake in Man. Diabetes.

[88] Laplante M., Sabatini D.M. (2012). mTOR Signaling in Growth Control and Disease. Cell.

[89] Chen H., Nie Q., Hu J., Huang X., Yin J., Nie S. (2021). Multiomics Approach to Explore the Amelioration Mechanisms of Glucomannans on the Metabolic Disorder of Type 2 Diabetic Rats. J. Agric. Food Chem..

[90] Zalewski B.M., Chmielewska A., Szajewska H. (2015). The effect of glucomannan on body weight in overweight or obese children and adults: A systematic review of randomized controlled trials. Nutrition.

[91] Keithley J.K., Swanson B., Mikolaitis S.L., DeMeo M., Zeller J.M., Fogg L., Adamji J. (2013). Safety and Efficacy of Glucomannan for Weight Loss in Overweight and Moderately Obese Adults. J. Obes..

[92] Arvill A., Bodin L. (1995). Effect of short-term ingestion of konjac glucomannan on serum cholesterol in healthy men. Am. J. Clin. Nutr..

[93] Lei P., Chen H., Ma J., Fang Y., Qu L., Yang Q., Peng B., Zhang X., Jin L., Sun D. (2022). Research progress on extraction technology and biomedical function of natural sugar substitutes. Front. Nutr..

[94] Bonelli A., Menna P., Minotti G., Angeletti S., Comandini A., Picollo R., Quarchioni E., Russo V., Salvatori E., Ferravante F. (2021). Safety and tolerability of a novel oral nutritional supplement in healthy volunteers. Clin. Nutr..

[95] Folwarski M., Kłęk S., Zoubek-Wójcik A., Szafrański W., Bartoszewska L., Figuła K., Jakubczyk M., Jurczuk A., Kamocki Z., Kowalczyk T. (2022). Foods for Special Medical Purposes in Home Enteral Nutrition-Clinical Practice Experience. Multicenter Study. Front. Nutr..

[96] Zhang W., Ren X., Zhang L., Chen J. (2022). Preparation and Performance of Thickened Liquids for Patients with Konjac Glucomannan-Mediated Dysphagia. Molecules.

[97] Cichero J.A.Y., Steele C., Duivestein J., Clavé P., Chen J., Kayashita J., Dantas R., Lecko C., Speyer R., Lam P. (2013). The Need for International Terminology and Definitions for Texture-Modified Foods and Thickened Liquids Used in Dysphagia Management: Foundations of a Global Initiative. Curr. Phys. Med. Rehabil. Rep..

[98] Tatirat O. (2012). Physicochemical properties of extrusion-modified konjac glucomannan. Carbohydr. Polym..

[99] Chen M., Wang H., Yan Q., Zheng Q., Yang M., Lv Z., He M., Feng L., Zhao J., Tang T. (2016). Effects of dietary oxidized konjac glucomannan sulfates (OKGMS) and acidolysis-oxidized konjac glucomannan (A-OKGM) on the immunity and expression of immune-related genes of Schizothorax prenanti. Fish Shellfish Immunol..

[100] Zhang L. (2017). Effects of oxidized konjac glucomannan on the intestinal microbial flora and intestinal morphology of Schizothorax prenanti. Aquacult. Int..

[101] Zheng Q., Wu Y., Xu H. (2015). Effect of dietary oxidized konjac glucomannan on Schizothorax prenanti growth performance, body composition, intestinal morphology and intestinal microflora. Fish Physiol. Biochem..

[102] Liu M., Zou T., Li H., Zhou M., Cheng Z. (2021). Research Status of the Technology for Reducing Sulfur Dioxide Residue in Konjac Flour. Farm Prod. Process..

[103] Vuksan V., Sievenpiper J.L., Xu Z., Wong E.Y.Y., Jenkins A.L., Beljan-Zdravkovic U., Leiter L.A., Josse R.G., Stavro M.P. (2001). Konjac-Mannan and American Ginsing: Emerging Alternative Therapies for Type 2 Diabetes Mellitus. J. Am. Coll. Nutr..

[104] Deng J., Zhong J., Long J., Zou X., Wang D., Song Y., Zhou K., Liang Y., Huang R., Wei X. (2020). Hypoglycemic effects and mechanism of different molecular weights of konjac glucomannans in type 2 diabetic rats. Int. J. Biol. Macromol..

